# Predictive value of pretreatment MRI texture analysis in patients with primary nasopharyngeal carcinoma

**DOI:** 10.1007/s00330-018-5961-6

**Published:** 2019-01-07

**Authors:** Jiaji Mao, Jin Fang, Xiaohui Duan, Zehong Yang, Minghui Cao, Fang Zhang, Liejing Lu, Xiang Zhang, Xiaoyan Wu, Yue Ding, Jun Shen

**Affiliations:** 1 Department of Radiology, Sun Yat-Sen Memorial Hospital, Sun Yat-Sen University, No. 107 Yanjiang Road West, Guangzhou, 510120 People’s Republic of China; 2 Guangdong Provincial Key Laboratory of Malignant Tumor Epigenetics and Gene Regulation, Medical Research Center, Sun Yat-Sen Memorial Hospital, Sun Yat-Sen University, No. 107 Yanjiang Road West, Guangzhou, 510120 People’s Republic of China; 3 Medical Imaging Center, The First Affiliated Hospital, Jinan University, No. 613 Huangpu Road West, Guangzhou, 510632 People’s Republic of China

**Keywords:** Magnetic resonance imaging, Nasopharyngeal neoplasms, Prognosis, Risk factors, Radiomics

## Abstract

**Objectives:**

To determine the predictive value of pretreatment MRI texture analysis for progression-free survival (PFS) in patients with primary nasopharyngeal carcinoma (NPC).

**Methods:**

Ethical approval by the institutional review board was obtained for this retrospective analysis. In 79 patients with primary NPC, texture analysis of the primary tumour was performed on pretreatment T2 and contrast-enhanced T1-weighted images (T2WIs and CE-T1WIs). The Cox proportional hazards model was used to determine the association of texture features, tumour volume and the tumour-node-metastasis (TNM) stage with PFS. Survival curves were plotted using the Kaplan-Meier method. The prognostic performance was evaluated with the receiver operating characteristic (ROC) analyses and C-index.

**Results:**

Tumour volume (hazard ratio, 1.054; 95% confidence interval [CI], 1.016–1.093) and CE-T1WI-based uniformity (hazard ratio, 0; 95% CI, 0–0.001) were identified as independent predictors for PFS (*p* < 0.05). Kaplan-Meier analysis showed that smaller tumour volume (less than the cut-off value, 11.699 cm^3^) and higher CE-T1WI-based uniformity (greater than the cut-off value, 0.856) were associated with improved PFS (*p* < 0.05). The combination of CE-T1WI-based uniformity with tumour volume and the overall stage predicted PFS better (area under the curve [AUC], 0.825; Cindex, 0.794) than the tumour volume (AUC, 0.659; C-index, 0.616) or the overall stage (AUC, 0.636; C-index, 0.627) did (*p <* 0.05).

**Conclusions:**

A texture parameter of pretreatment CE-T1WI-based uniformity improves the prediction of PFS in NPC patients.

**Key Points:**

*• Higher CE-T1WI-based uniformity and smaller tumour volume are predictive of improved PFS in NPC patients.*

• *The combination of CE-T1WI-based uniformity with tumour volume and the overall stage has a better predictive ability for PFS than the tumour volume or the overall stage alone.*

• *Pretreatment MRI texture analysis has a prognostic value for NPC patients.*

**Electronic supplementary material:**

The online version of this article (10.1007/s00330-018-5961-6) contains supplementary material, which is available to authorized users.

## Introduction

Nasopharyngeal carcinoma (NPC) is a cancer arising from the nasopharynx epithelium with a very unique geographic distribution [1]. It is one of the most common malignant tumours in South-Eastern China, South-Eastern Asia and Northern Africa [2]. Despite advances in radiotherapy and chemotherapy, locoregional recurrence and distant metastasis can occur in nearly 10–15% patients during the first 2 years after the start of treatment and only 72.9% patients have a 2-year progression-free survival (PFS) [3]. TNM staging system is insufficient to predict the prognosis of NPC, as NPC patients with the same TNM stage often show different clinical outcomes [4]. Several molecular biomarkers have been correlated with survival in NPC patients [5, 6]. Nonetheless, these biomarkers are obtained through randomly sampled biopsy that evaluates a small fraction of the tumour. As such, they have inherent limitations including the evaluation of invasiveness and misrepresentation of the entire tumour due to heterogeneity [7].

High-throughput extraction of quantitative features from images is an attractive strategy for objective assessment of tumour heterogeneity [8]. Texture features that analyse the distribution and relationship of pixel or voxel grey levels within the images are most widely used [9]. In previous studies, texture features extracted from images of PET, CT or MRI have been associated with clinical prognosis in various types of cancers [10–13]. However available data for NPC are scarce. Only a recent study reported that MRI images radiomics features could be used to predict PFS in patients with advanced NPC [14]. However, this study only investigated NPC patients with advanced disease (stages III–IV). In addition, radiomics-based nomograms are difficult to interpret and time-consuming to be used in daily practice. Furthermore, tumour volume has been shown to be a very important prognostic factor for NPC patients [15, 16]. Whether texture features combined with tumour volume and TNM stage can provide a better prognostic ability for NPC patients remains unknown.

The purpose of our study was to determine the predictive value of pretreatment MRI texture analysis for PFS in patients with primary NPC. To achieve this aim, we performed texture analysis of pretreatment T2-weighted images (T2WIs) and contrast-enhanced T1-weighted images (CE-T1WIs), and combined it with tumour volume and TNM stage.

## Materials and methods

### Patients

Institutional research ethics board approval was obtained, and written informed consent was waived for this retrospective study. Consecutive patients with NPC, who underwent MRI between October 2012 to August 2014, were identified from the Picture Archiving and Communication System of our institution. The inclusion criteria were (a) patients with primary NPC that was confirmed by histological biopsy, (b) patients who underwent nasopharyngeal-neck MRI within 2 weeks before treatment and (c) patients who achieved a complete response after treatment. A total of 116 patients met the inclusion criteria. Patients were excluded if they (a) had other malignancies (*n* = 3) or distant metastases at diagnosis (*n* = 1), (b) had a primary tumour merely confined to nasopharyngeal mucous but without mass formation (*n* = 17) or (c) had incomplete follow-up data (*n* = 16). Finally, 79 NPC patients (52 men and 27 women; mean age, 46.6; age range, 15–73 years) were included in this study.

### Treatment and follow-up

All patients received two-dimensional radiotherapy, three-dimensional conformal radiotherapy or intensity-modulated radiation therapy to treat the primary tumour and cervical adenopathy. The total radiation doses ranged from 66 to 75 Gy (mean, 70 Gy). Stage I patients had not received chemotherapy and stages II to IV patients received neoadjuvant or adjuvant chemotherapy and/or concurrent chemotherapy with radiotherapy, according to the National Comprehensive Cancer Network clinical practice guidelines for NPC [17, 18]. Patients were followed up every 3 months in the first to second year, then every 6 months in the third to fifth year and once a year thereafter. In each visit, medical history, physical examination, nasopharyngoscopy, nasopharyngeal-neck MRI, thoracic CT, abdominal sonography and whole-body bone scintigraphy were performed. All follow-ups ended in January 2018.

### Definition of outcomes

To avoid extended follow-up, PFS was chosen as the endpoint. PFS was calculated from the start of treatment to the date of disease progression (locoregional recurrences or distant metastases), death from any cause or the date of the last follow-up visit, whichever occurred first. The minimum follow-up time to ascertain survival without progression was 24 months. All local relapses were diagnosed by nasopharyngoscopy with biopsy and MRI that showed progressive bone erosion and/or soft tissue swelling. Regional recurrences were diagnosed by fine needle aspiration biopsy when clinical examination of the neck and MRI that showed progressive cervical adenopathy. Notably, cervical lymph nodes were suspicious for residual metastasis when these persistent lymph nodes do not regress completely by 3 months after completion of treatment, either radiotherapy or concomitant chemotherapy and radiotherapy. When cervical lymph nodes reappear and progress after a period of initial regression, the presence of regional recurrence was suspected. In these cases, fine needle aspiration biopsy was performed to confirm the presence of recurrence. If the cervical lymph nodes were the only site with disease, these residual and recurrent nodes were eradicated by salvage surgical neck dissection. Distant metastases were diagnosed based on clinical symptoms, physical examination and imaging modalities including nasopharyngeal-neck MRI, thoracic CT, abdominal sonography and whole-body bone scintigraphy.

### MRI

MRI was performed by using a 3.0 T scanner (Achieva, Philips Healthcare) with a 16-channel head-neck synergic coil. The acquisition sequences consisted of axial turbo spin echo (TSE) T2-weighted imaging, axial and sagittal TSE T1-weighted imaging and coronal short time inversion recovery imaging. Axial and sagittal contrast-enhanced TSE T1-weighted imaging was obtained after intravenous bolus injection of gadopentetate dimeglumine (Magnevist, Bayer Schering) was administered at a dosage of 0.1 mmol/kg of body weight. Imaging sequences and acquisition parameters are listed in Supplementary Table 1. Postcontrast T1WIs were obtained using the same parameters as the precontrast T1WIs except for using the fat-suppression technique.

### Image analysis

**Fig. 1:**
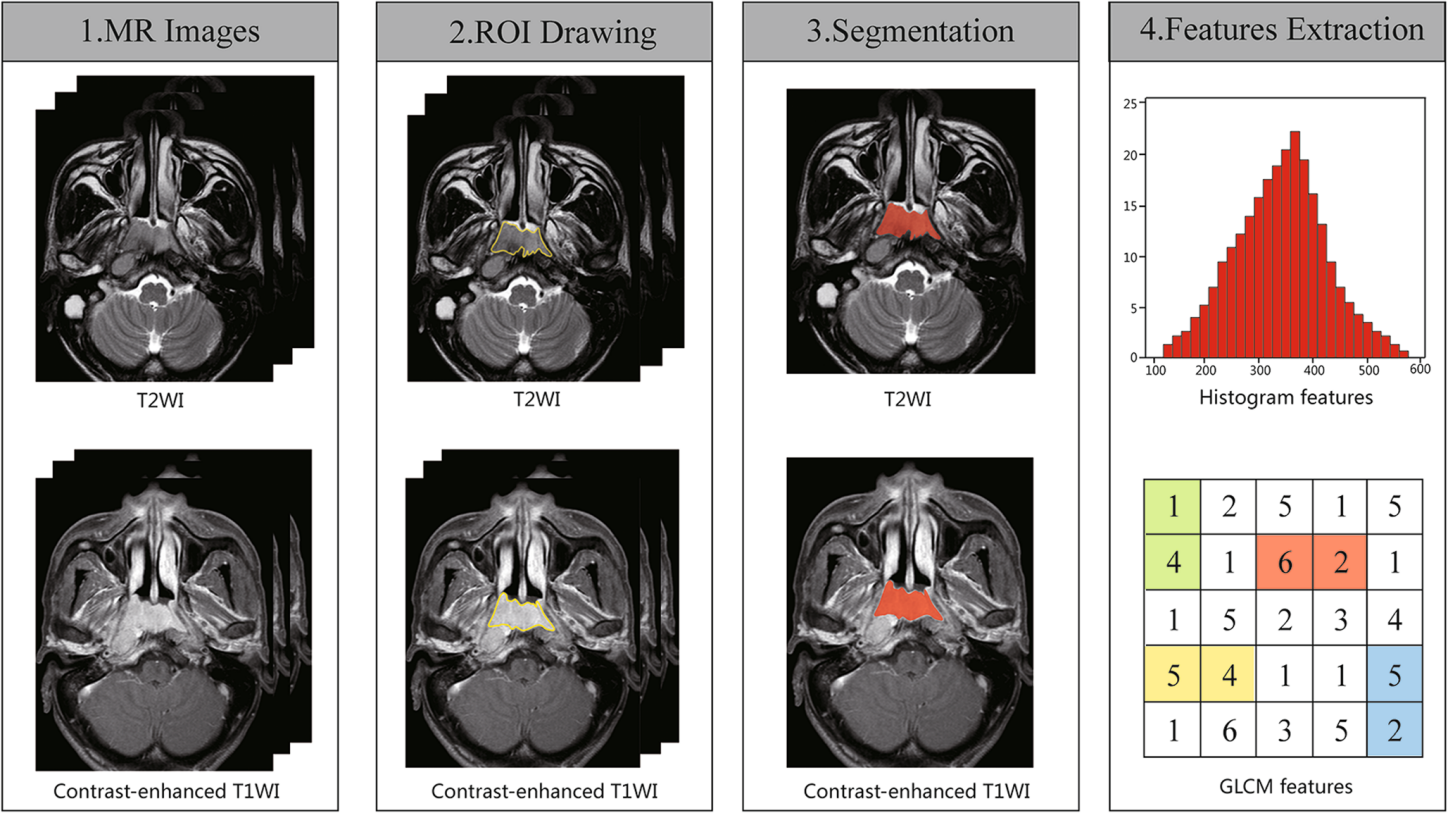
Schematic diagram for data processing

Two radiologists with 6 years (J.F.) and 10 years (X.D.) of experience in diagnostic MRI assessed all images for each patient and staged the tumour according to the established staging system by consensus [19]. For the texture analysis, all axial T2WIs and CE-T1WIs were individually transferred to the in-house texture analysis software package (Omni-Kinetics Version 2.1, GE Healthcare) [20]. A free-hand region of interest (ROI) was manually drawn to delineate the whole tumour on each slice by a radiologist with 6 years of experience (J.M.). Three-dimensional segmentation of the tumours was then completed automatically by the Omni-Kinetics software package. Each segmentation was checked and validated by a senior radiologist with 23 years of experience (J.S.). The obtained volume of interest for each lesion was executed, and values of texture features were calculated. The first-order and second-order texture features were then obtained by using histogram analysis and grey-level co-occurrence matrix (GLCM), respectively. Five first-order texture parameters (histogram features) including entropy, uniformity, variance, skewness and kurtosis, together with four second-order texture parameters (GLCM features) including GLCM entropy, angular second moment (ASM), cluster prominence and cluster shade were chosen to measure NPC heterogeneity in our study. The texture features included are described in detail in Supplementary Table 2. The schematic diagram for data processing is shown in Fig. 1.

### Statistical analysis

All numerical data are presented as the mean ± standard deviation. The effects of texture parameters, tumour volume, T stage, N stage, overall stage, age and gender on PFS were analysed using the Cox proportional hazards regression model. The individual effect of each variable on PFS was first tested by using univariate analyses; then, multivariate Cox proportional hazards regression analysis was performed to determine the independent predictors for PFS. Patients were classified into high-risk or low-risk groups according to independent predictors, and the thresholds of predictors were determined by the Youden index that maximised both sensitivity and specificity in the receiver operating characteristic (ROC) curves. Survival curves of the high- and low-risk groups were plotted using the Kaplan-Meier method (log-rank test). All these statistical analyses were performed using SPSS software version 22.0 (SPSS Inc). The abilities of survival models involving each independent predictor or combined predictors in predicting 2-year PFS were assessed by the ROC analysis using the R software (version 3.2.4; R Foundation for Statistical Computing) with the pROC package. The area under the curve (AUC), sensitivity, specificity, positive predictive value and negative predictive value of each model were calculated. The AUCs were compared across models by using the Hanley and McNeill test. Prognostic performances of survival models were evaluated with C-index using the R software (version 3.2.4; R Foundation for Statistical Computing) with the Hmisc and Survival packages. The C-indices were compared by using the Z-score test with the “compareC” package in R software [21]. All statistical analyses were two-sided, and a *p* value of less than 0.05 was considered as statistically significant.

## Results

### Patient characteristics and survival outcome

Baseline clinicopathologic characteristics of 79 NPC patients are summarised in Table 1. Twenty-six patients had recurrences (10 locoregional relapses and 16 distant metastases) with a PFS mean of 12.2 months (range, 4–26 months). Among the 10 patients with locoregional relapses, there were 9 patients with local recurrence and 1 patient with regional relapse. The diagnosis of this patient with regional relapse who showed progressive cervical adenopathy on MRI was subsequently confirmed by fine needle aspiration biopsy. Fifty-three patients had no tumour recurrence with a PFS mean of 30.2 months (range, 24–47 months).

**Table 1:** Clinicopathologic characteristics of 79 NPC patients

Clinical profiles	*n*
Age (years)	46.6 ± 11.5
Sex	
Male	52 (65.8%)
Female	27 (34.2%)
Pathologic type^a^	
Keratinizing squamous cell carcinoma	1 (1.3%)
Differentiated non-keratinizing carcinoma	6 (7.6%)
Undifferentiated non-keratinizing carcinoma	72 (91.1%)
T stage^b^	
T1	35 (44.3%)
T2	19 (24.1%)
T3	15 (19.0%)
T4	10 (12.7%)
N stage^b^	
N0	4 (5.1%)
N1	32 (40.5%)
N2	27 (34.2%)
N3	16 (20.3%)
Overall stage^b^	
I	3 (3.8%)
II	22 (27.8%)
III	30 (38.0%)
IV	24 (30.4%)
Progression-free survival (months)	
Mean (range)	24.7 (4–47)

^a^According to the 2005 World Health Organisation classification of tumours

^b^According to the 7th UICC/AJCC staging system

### Predictors for PFS

**Fig. 2:**
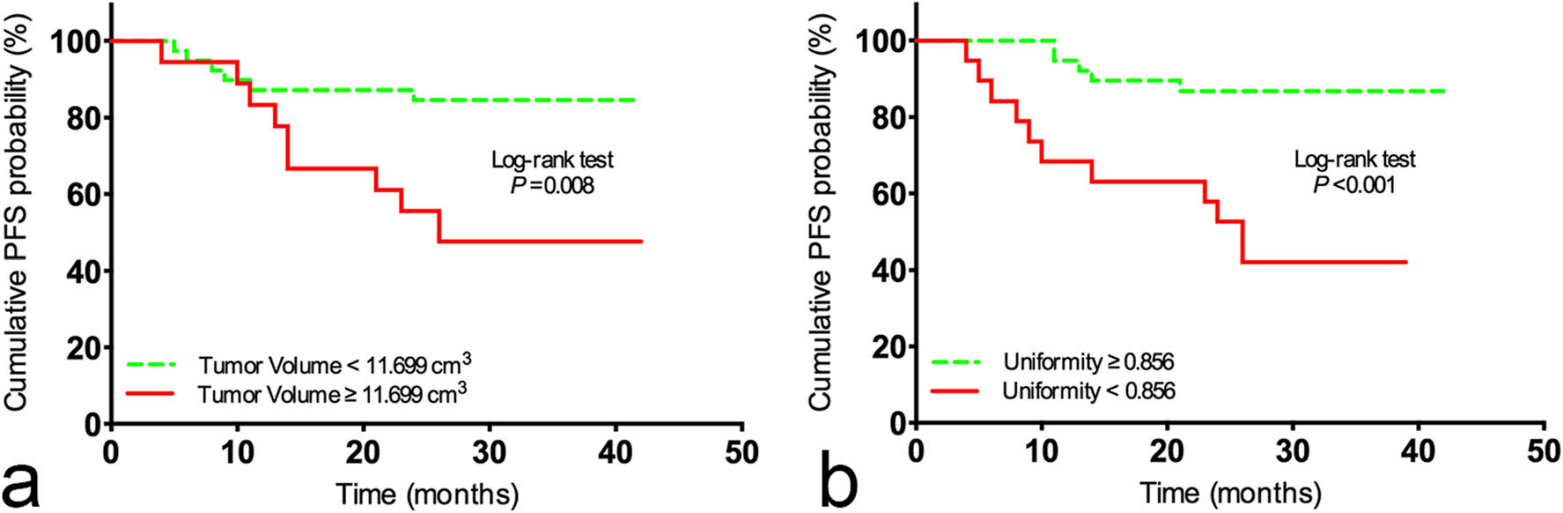
Kaplan-Meier plots of NPC patients stratified according to (**a**) tumour volume and (**b**) uniformity measured on CE-T1WI showed a significant difference for PFS (progression-free survival) with log-rank *p* values of 0.008 and < 0.001, respectively

**Fig. 3:**
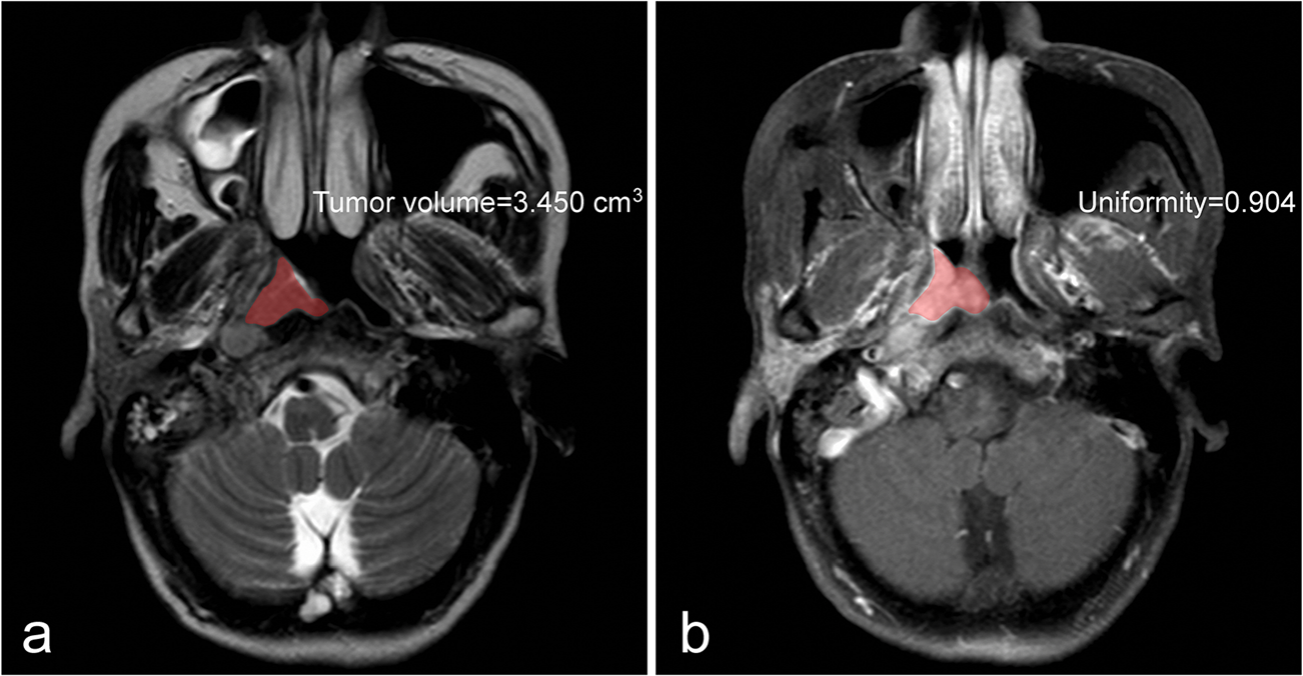
A 56-year-old woman with undifferentiated non-keratinizing NPC (T2N3bM0). **a** Tumour volume measured on the axial T2WI was less than the cut-off value (11.699 cm^3^) and classified as the low-risk group. **b** Uniformity measured on axial CE-T1WI was higher than the cut-off value (0.856) and identified as low-risk group. During 38 months of follow-up, there was no evidence of locoregional relapse or distant metastasis

**Fig. 4:**
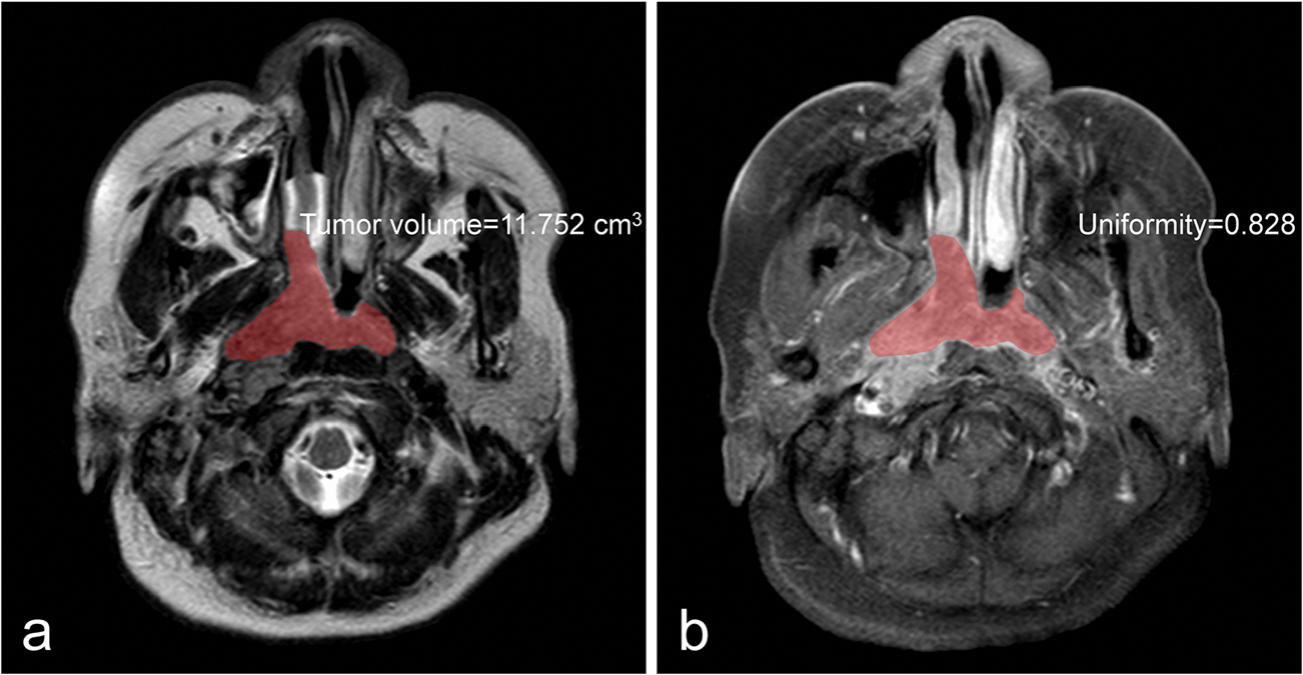
A 46-year-old woman with undifferentiated, non-keratinizing NPC (T2N3aM0). **a** Tumour volume measured on the axial T2WI was larger than the cut-off value (11.699 cm^3^) and classified as high-risk group. **b** Uniformity measured on CE-T1WI was less than the cut-off value (0.856) and identified as high-risk group. Fourteen months after treatment, the patient had liver metastases

Values of texture parameters are summarised in Supplementary Table 3. Univariate Cox regression analyses showed that T stage, overall stage, tumour volume, T2WI-based GLCM entropy, as well as CE-T1WI-based uniformity, kurtosis and angular second moment were associated with PFS (*p* = 0.004, *p* = 0.017, *p* = 0.011, *p* = 0.011, *p* < 0.001, *p* = 0.017, *p* = 0.026, respectively) (Table 2). Further multivariate analysis showed that tumour volume (hazard ratio, 1.054; 95% CI, 1.016–1.093) and CE-T1WI-based uniformity (hazard ratio, 0; 95% CI, 0–0.001) were independent predictors for PFS (*p* = 0.005, *p* = 0.001) (Table 3). According to the ROC curves and the Youden index, the optimal cut-off values for tumour volume and uniformity were 11.699 cm^3^ and 0.856, respectively. Kaplan-Meier curves showed that less tumour volume (less than the cut-off value, 11.699 cm^3^) and higher CE-T1WI-based uniformity (equal to or higher than the cut-off value, 0.856) were associated with improved PFS (Fig. 2). Two representative cases are shown in Fig. 3 and Fig. 4.

**Table 2:** Univariate Cox proportional hazard analyses of variables associated with PFS

Variable	PFS	
	Hazard ratio (95% confidence interval)	*p* value
Age (y)	1.031 (0.994, 1.069)	0.103
Sex (male vs female)	0.379 (0.143, 1.006)	0.051
T stage (1–2 vs 3–4)	3.087 (1.422, 6.700)	0.004*
N stage (0–1 vs 2–3)	1.766 (0.786, 3.970)	0.169
Overall stage (I–II vs III–IV)	4.357 (1.303, 14.563)	0.017*
Tumour volume (cm^3^)	1.045 (1.013, 1.079)	0.011*
T2WI-based texture parameters		
Entropy	0.706 (0.203, 2.454)	0.584
Uniformity	0.037 (0.000, 9.422)	0.242
Variance	1 (1.000, 1.001)	0.105
Skewness	1.159 (0.619, 2.172)	0.645
Kurtosis	1.038 (0.902, 1.193)	0.603
GLCM entropy	0.607 (0.409, 0.902)	0.011*
Angular second moment	3.627 × 10^55^ (0, 0.001)	0.765
Cluster prominence	1 (0.999, 1.000)	0.799
Cluster shade	1 (0.999, 1.000)	0.980
CE-T1WI-based texture parameters		
Entropy	7.484 (0.981, 57.078)	0.050
Uniformity	0 (0, 0.002)	< 0.001*
Variance	1 (0.999, 1.000)	0.997
Skewness	1.391 (0.614, 3.152)	0.429
Kurtosis	0.558 (0.341, 0.913)	0.017*
GLCM entropy	0.692 (0.442, 1.082)	0.100
Angular second moment	0 (0, 8.938)	0.026*
Cluster prominence	1 (0.999, 1.000)	0.187
Cluster shade	1 (0.999, 1.000)	0.812

*PFS* progression-free survival, *T2WI* T2-weighted image, *CE-T1WI* contrast-enhanced T1-weighted image, *GLCM* grey-level co-occurrence matrix

*p < 0.05

**Table 3:** Multivariate Cox proportional hazard analysis of PFS

Variable	PFS	
	Hazard ratio (95% confidence interval)	*p* value
T stage (1–2 vs 3–4)	–	0.789
Overall stage (I–II vs III–IV)	–	0.243
Tumour volume	1.054 (1.016, 1.093)	0.005*
T2WI-based texture parameters		
GLCM entropy	–	0.319
CE-T1WI-based texture parameters		
Uniformity	0 (0, 0.001)	0.001*
Kurtosis	–	0.551
Angular second moment	–	0.692

*PFS* progression-free survival, *T2WI* T2-weighted image, *CE-T1WI* contrast-enhanced T1-weighted image, *GLCM* grey-level co-occurrence matrix

*p < 0.05

### Prognostic performance of survival models

Although the T stage, N stage or overall stage were not independent predictors of PFS, the TNM staging system is widely used to predict the overall outcome. Thus, we included the overall stage in the analysis. The results of ROC analyses of single texture parameter, tumour volume and overall stage or combined in predicting 2-year PFS are summarised in Table 4. The AUC of CE-T1WI-based uniformity was higher than those of the overall stage and tumour volume. The AUC of the combination of CE-T1WI-based uniformity with overall stage and tumour volume was 0.825, which was higher than those of the overall stage and tumour volume (*p* = 0.014; *p* = 0.021). Prognostic performances of different survival models are shown in Table 5. The C-index of CE-T1WI-based uniformity was higher than those of the overall stage and tumour volume. When combined with overall stage or tumour volume, CE-T1WI-based uniformity predicted PFS better than the overall stage or tumour volume alone did (*p* = 0.046; *p* = 0.043). Combination of CE-T1WI-based uniformity with overall stage and tumour volume yielded the highest C-index, and thus, showed better predictive performance than overall stage or tumour volume alone did (*p* = 0.006; *p* = 0.009).

**Table 4:** ROC analyses of different models in predicting 2-year progression-free survival

Models	AUC	SEN (%)	SPE (%)	PPV (%)	NPV (%)
CE-T1WI-based uniformity	0.710 (0.530, 0.889)	76.2	66.7	86.5	50.0
		(60.2, 87.2)	(38.7, 98.0)	(70.4, 94.9)	(27.9, 72.1)
Overall stage	0.636 (0.519, 0.752)	40.5	86.7	89.5	34.2
		(26.0, 56.7)	(58.4, 97.7)	(65.5, 98.2)	(20.1, 51.4)
Tumour volume	0.659 (0.472, 0.846)	78.6	60.0	84.6	50.0
		(62.8, 89.2)	(32.9, 82.5)	(68.8, 93.6)	(26.8, 73.2)
CE-T1WI-based uniformity + overall stage	0.540 (0.365, 0.715)	40.5	86.7	89.5	34.2
		(26.0, 56.7)	(58.4, 97.7)	(65.5, 98.2)	(20.1, 51.4)
CE-T1WI-based uniformity + tumour volume	0.784 (0.625, 0.943)	95.2	60.0	87.0	81.8
		(82.6, 99.2)	(32.9, 82.5)	(73.1, 94.6)	(47.8, 96.8)
CE-T1WI-based uniformity + overall stage + tumour volume	0.825 (0.696, 0.955)	85.7	73.3	90.0	64.7
		(70.8, 94.1)	(44.8, 91.1)	(75.4, 96.7)	(38.6, 84.7)

*CE-T1WI* contrast-enhanced T1-weighted image, *AUC* area under the curve, *SEN* sensitivity, *SPE* specificity, *PPV* positive predictive value, *NPV* negative predictive value

Data in parentheses are 95% confidence intervals (CIs)

**Table 5:** Prognostic performance of different models

Model	C-index	95% confidence interval
CE-T1WI-based uniformity	0.684	(0.531, 0.837)
Overall stage	0.627	(0.500, 0.754)
Tumour volume	0.616	(0.463, 0.769)
CE-T1WI-based uniformity + overall stage	0.756	(0.603, 0.909)
CE-T1WI-based uniformity + tumour volume	0.754	(0.601, 0.907)
CE-T1WI-based uniformity + overall stage + tumour volume	0.794	(0.641, 0.947)

*CE-T1WI* contrast-enhanced T1-weighted image

## Discussion

Our study demonstrated that tumour volume and CE-T1WI-based uniformity were independent predictors for PFS in patients with NPC. Specifically, higher CE-T1WI-based uniformity and smaller tumour volume were prognostic factors for favourable PFS. A single texture parameter, CE-T1WI-based uniformity, when combined with tumour volume and the overall stage, showed higher predictive ability (AUC, 0.825) than the tumour volume (AUC, 0.659) or the overall stage alone (AUC, 0.636). Texture analysis can improve PFS prediction when combined with clinical indexes, such as tumour volume or overall stage.

Identification of high-risk patients would be beneficial, like inviting to more intensive observation and/or more aggressive treatment [22]. High tumour heterogeneity is usually associated with poor prognosis [23]. Texture analysis allows for objective assessment of heterogeneity beyond visual interpretation [24]. Statistical texture analysis techniques have been the most widely used method, which can yield three orders of texture parameters [9]. The first-order texture parameters are obtained from the histogram of pixel intensity values, including uniformity (measure of homogeneity of the distribution of grey levels), skewness (measure of asymmetry of the pixel histogram) and kurtosis (measure of peakness of the pixel histogram), which describe the image grey-level heterogeneity [25]. The grey-level co-occurrence matrix (GLCM) measurement is a well-known second-order statistics method, which is calculated using spatial grey-level dependence matrices, and measures local heterogeneity related only to the neighbouring pixels, yielding texture parameters such as GLCM entropy (measure of randomness of the GLCM) and angular second moment (measure of homogeneity of the GLCM) [26]. The third-order statistics reveals the spatial relationship among three or more pixels [26]. A recent study showed that among 177 radiomics features including intensity, shape and texture features, many radiomics features were redundant [27]. In our study, five first-order parameters together with four GLCM parameters were selected for simplicity.

Previously, texture features have been useful in predicting prognosis of many types of cancers, such as oesophageal, head-and-neck, colorectal, breast and non-small cell lung cancer [10–13]. For NPC, intratumour heterogeneity can be assessed by texture features measured on the PET component of ^18^F-FDG PET/CT, and uniformity and skewness were found to be superior to traditional PET parameters in predicting clinical outcomes in patients with primary NPC [28]. Compared with PET/CT, MRI is more widely used in clinical workup to diagnose and stage NPC before treatment due to its excellent spatial resolution, absence of radiation and lower cost. In a recent study of 118 NPC patients, radiomics-based nomograms from pretreatment MRI were found to be useful prognostic predictors [14]. In this study, a total of 970 radiomics features were derived from T2WIs and CE-T1WIs in advanced NPC patients (stages III–IV). The prognostic ability of this MRI-based radiomics nomograms remains to be evaluated in low-stage NPC patients (stages I–II). In addition, only eight features of 970 radiomics features were found to be prognostic [14]. In the present study, 79 patients with stages I–IV NPC were enrolled. We assessed texture features derived from T2WIs and CE-T1WIs together with tumour volume. Our results demonstrated that a single texture parameter, CE-T1WI-based uniformity, was an independent factor for PFS in NPC patients. Moreover, both tumour volume and CE-T1WI-based uniformity could predict PFS in NPC patients with a comparable prognostic ability. Comparatively, a single texture parameter would be more favourable for clinical application than radiomics-based nomograms. Notably, when combined either with tumour volume or with the overall stage, CE-T1WI-based uniformity can yield a higher C-index for predicting PFS (C-index, 0.754 and 0.756, respectively), which is very close to the C-index attained by previously reported radiomics-based nomograms (C-index, 0.756) [14]. Moreover, when combined with both tumour volume and overall stage, the prognostic ability was further improved (AUC, 0.825; C-index, 0.794). These results suggest that CE-T1WI-based uniformity alone has a high prognostic performance. It can be used as a complementary index to improve the prognostic ability of frequent clinical indexes, such as tumour volume and overall stage.

In our study, higher CE-T1WI-based uniformity was a predictive factor for favourable PFS in patients with NPC; a more homogeneous enhancement with a tumour on CE-T1WI was associated with better prognosis. Similar results were also found in other cancers [29, 30]. For example, lung cancer with less heterogeneity on CE-T1WI (less entropy) is associated with improved 2-year PFS [31]. Higher uniformity based on contrast-enhanced CT is associated with improved survival in oesophageal cancer treated with chemotherapy and radiation therapy [32]. More homogeneity on contrast-enhanced radiographic images suggests more homogeneous angiogenesis within the tumour, associated with better prognosis in head and neck squamous cell carcinoma [33, 34]. Taken together, texture analysis of postcontrast images offers a non-invasive and low-cost new insight into the relationship between angiogenesis and patient survival.

In our study, larger tumour volume was shown to be an adverse predictor for PFS in NPC patients. It has been reported that larger volume is associated with poorer prognosis in many solid tumours, such as NPC, cholangiocarcinoma and tongue carcinoma [16, 35, 36]. Larger solid tumours are more likely to release more cells, exposing to metastases and poor prognosis [37].

Our study still has some limitations. First, the sample size, in particular for the recurrent cases, is relatively small because patients were enrolled from a single centre. The prognostic value of uniformity remains to be validated in a large cohort study. Second, the follow-up period was relatively short. Only 2 years PFS was adopted as the endpoint of survival outcome. Long-term overall survival was not attempted in our study because most recurrences or metastasis occur within 2 years after primary treatment in NPC patients [38]. Third, NPC patients were treated by concurrent radiotherapy and chemotherapy or radiotherapy alone; and varying doses of radiation were adopted for radiotherapy regimen. These different strategies might be confounding factors for the evaluation of PFS.

In conclusion, our study showed that tumour volume and pretreatment CE-T1WI-based uniformity are prognostic predictors in NPC patients. A small size and homogeneous contrast-enhancement with a primary lesion are predictive of better PFS. The addition of CE-T1WI-based texture analysis to tumour volume and overall stage can improve the prediction of PFS in NPC patients.

## Electronic supplementary material

ESM 1 (DOC 817 kb)

## Abbreviations

AUC: Area under the curve
CE-T1WI: Contrast-enhanced T1-weighted image
CI: Confidence interval
GLCM: Grey-level co-occurrence matrix
NPC: Nasopharyngeal carcinoma
PFS: Progression-free survival
ROC: Receiver operating characteristic
T2WI: T2-weighted image
