# Supplementary material for: Predictive value of pretreatment MRI texture analysis in patients with primary nasopharyngeal carcinoma
Source: Eur Radiol. 2019 Jan 7;29(8):4105–13. doi: 10.1007/s00330-018-5961-6 (PMC6610272; doi:10.1007/s00330-018-5961-6)
Supplement: Supplementary file 1 — (DOC 817 kb) [file 330_2018_5961_MOESM1_ESM.doc]

**Supplementary Materials**

Table 1. Imaging sequences and acquisition parameters of MRI

| **Sequence** | **Slice orientation** | **TR/TE (ms)** | **TI**  **(ms)** | **Slice**  **number** | **Slice thickness**  **/gap (mm)** | **FOV (mm^2^)** | **Matrix** | **Fat-suppression** |
| --- | --- | --- | --- | --- | --- | --- | --- | --- |
| TSE T2WI | transverse | 2579/90 | / | 36 | 5/1 | 230× 230 | 288 × 194 | No |
| TSE T1WI | transverse | 532/18 | / | 36 | 5/1 | 230× 230 | 288 × 220 | No |
| TSE T1WI | sagittal | 550/20 | / | 15 | 5/1 | 260×230 | 372 × 262 | No |
| T2W STIR | coronal | 3000/60 | 220 | 15 | 5/1 | 200×250 | 248 × 233 | Yes |

Note. TSE: turbo spin echo; T2WI: T2-weighted imaging; T1WI: T1-weighted imaging; STIR: short time inversion recovery; TR: repetition time; TE: echo time; TI: inversion time; FOV: field of view.

**Table 2**. Definitions of texture features

| Texture features | Definition | Formula |
| --- | --- | --- |
| **First-order texture parameters** |  |  |
| Entropy | Measure of randomness of the distribution of grey levels | 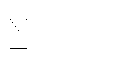 |
| Uniformity | Measure of homogeneity of the distribution of grey levels | 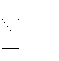 |
| Variance | Measure of dispersion of distribution of grey levels | 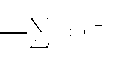 |
| Skewness | Measure of asymmetry of the pixel histogram | 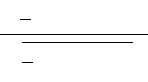 |
| Kurtosis | Measure of peakness of the pixel histogram | 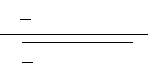 |
| **Second-order texture parameters** |  |  |
| GLCM Entropy | Measure of randomness of the Grey-Level Co-occurrence Matrix (GLCM) | 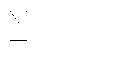 |
| Angular Second Moment | Measure of homogeneity of the GLCM | 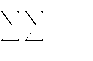 |
| Cluster Prominence | Measure of skewness and asymmetry of the GLCM | 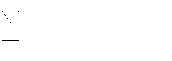 |
| Cluster Shade | Measure of skewness and uniformity of the GLCM | 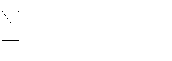 |

**Table 3**. Values of textural analysis features

| Features | Mean | SD | Min | Max |
| --- | --- | --- | --- | --- |
| **T2WI-based texture parameters** |  |  |  |  |
| Entropy | 6.649 | 0.311 | 5.765 | 7.231 |
| Uniformity | 0.802 | 0.061 | 0.564 | 0.890 |
| Variance | 1867.129 | 1211.832 | 657.794 | 8841.540 |
| Skewness | 0.282 | 0.604 | -1.009 | 1.686 |
| Kurtosis | 2.507 | 2.581 | -0.988 | 13.776 |
| GLCM Entropy | 10.531 | 0.927 | 8.726 | 12.123 |
| Angular Second Moment | 0.0009 | 0.0004 | 0.0002 | 0.0024 |
| Cluster Prominence | 27662.012 | 104814.973 | -242628 | 386873 |
| Cluster Shade | 47599368.4 | 31584663.5 | 365×10^4^ | 164×10^6^ |
| **CE-T1WI-based texture parameters** |  |  |  |  |
| Entropy | 6.891 | 0.255 | 6.343 | 7.439 |
| Uniformity | 0.865 | 0.042 | 0.681 | 0.924 |
| Variance | 2471.061 | 2151.123 | 610.746 | 14294.200 |
| Skewness | -0.718 | 0.529 | -1.441 | 1.142 |
| Kurtosis | 1.617 | 1.148 | -0.590 | 4.279 |
| GLCM Entropy | 10.425 | 1.157 | 7.749 | 12.020 |
| Angular Second Moment | 0.0011 | 0.0005 | 0.0003 | 0.0027 |
| Cluster Prominence | -219363.39 | 196305.149 | -845581 | 160584 |
| Cluster Shade | 87831578.9 | 56053450.8 | 158×10^5^ | 245×10^6^ |

Note. T2WI: T2-weighted imaging; CE-T1WI: contrast-enhanced T1-weighted imaging.
